# Moderators of ayahuasca’s biological antidepressant action

**DOI:** 10.3389/fpsyt.2022.1033816

**Published:** 2022-12-05

**Authors:** Geovan Menezes de Sousa, Vagner Deuel de Oliveira Tavares, Ana Cecília de Menezes Galvão, Raíssa Nóbrega de Almeida, Fernanda Palhano-Fontes, Bruno Lobão-Soares, Fúlvio Aurélio de Morais Freire, Emerson Arcoverde Nunes, João Paulo Maia-de-Oliveira, Daniel Perkins, Jerome Sarris, Dráulio Barros de Araujo, Nicole Leite Galvão-Coelho

**Affiliations:** ^1^Laboratory of Hormone Measurement, Department of Physiology and Behavior, Federal University of Rio Grande do Norte, Natal, RN, Brazil; ^2^Graduate Program in Psychobiology, Center for Biosciences, Federal University of Rio Grande do Norte, Natal, RN, Brazil; ^3^Brain Institute, Federal University of Rio Grande do Norte, Natal, RN, Brazil; ^4^Onofre Lopes University Hospital, Federal University of Rio Grande do Norte, Natal, RN, Brazil; ^5^National Science and Technology Institute for Translational Medicine, São Paulo, Brazil; ^6^Department of Biophysics and Pharmacology, Federal University of Rio Grande do Norte, Natal, RN, Brazil; ^7^Department of Clinical Medicine, Federal University of Rio Grande do Norte, Natal, RN, Brazil; ^8^School of Social and Political Science, University of Melbourne, Parkville, VIC, Australia; ^9^Psychae Institute, Melbourne, VIC, Australia; ^10^NICM Health Research Institute, Western Sydney University, Westmead, NSW, Australia; ^11^The Florey Institute of Neuroscience and Mental Health, University of Melbourne, Parkville, VIC, Australia

**Keywords:** psychedelics, cortisol, BDNF, inflammation, depression

## Abstract

**Introduction:**

The understanding of biological responses to psychedelics with antidepressant potential is imperative. Here we report how a set of acute parameters, namely emotional (depressive symptoms), cognitive (psychedelic experience), and physiological (salivary cortisol), recorded during an ayahuasca dosing session, modulated serum brain-derived neurotrophic factor (BDNF), serum cortisol (SC), serum interleukin 6 (IL-6), plasma C-reactive protein (CRP), and salivary cortisol awakening response (CAR).

**Methods:**

Results were analyzed 2 days after the psychedelic intervention (ayahuasca) versus placebo in both patients with treatment-resistant depression and healthy volunteers. These measures were assessed as part of a randomized double-blinded, placebo-controlled trial (*n* = 72).

**Results:**

Results revealed that larger reductions of depressive symptoms during the dosing session significantly moderated higher levels of SC in patients. Whereas lesser changes in salivary cortisol levels during the ayahuasca intervention were related to higher BDNF levels in patients with a larger clinical response in the reduction in depressive symptoms. No moderator was found for patient’s CAR, IL-6, and CRP responses to ayahuasca and for all biomarker responses to ayahuasca in healthy controls and in the placebo group.

**Discussion:**

In summary, some specific emotional and physiological parameters during experimental ayahuasca session were revealed as critical moderators of the improvement of major depression biomarkers, mainly BDNF and SC two days after ayahuasca intake. These findings contribute to paving the way for future studies investigating the biological antidepressant response to psychedelic therapy.

## Introduction

The classic serotonergic psychedelics such as lysergic acid diethylamide (LSD), mescaline, psilocybin, and DMT/ayahuasca (1–3) are a promising treatment for major depression disorder (MDD) (4–8), since even though the current use of antidepressant therapy offers clear benefits in clinical practice, many patients exhibit only a partial or no response (9, 10). Our recent index meta-analysis that investigated the effects of classic serotonergic psychedelics vs. placebo in depressive symptoms showed a large effect size of psychedelics in reduction of these symptoms in mid-term (2–7 days after treatment), whereas moderate effect sizes were found in the short (3 h–1 day) and longer-term (16–60 days after treatments) analysis (4).

Although the classic psychedelics promote changes in an array of neurobiological mechanisms, we have seen more studies integrating the psychedelic mental states and electrophysiological (11, 12) or neuroimage approaches (1, 13–17). However, a “multidimensional biological analysis” underlying depressive symptoms is imperative for the understanding of a well-characterized clinical response to psychedelics and to further help in the development of novel treatments. This thought is supported by the Research Domain Criteria (RDoC) of the National Institute of Mental Health (NIMH), which emphasizes on using different biological markers as tools for investigating mental disorders and their treatments (18). Even though, there are few studies that have investigated molecular biomarkers in response to classic psychedelics, most of them only concern healthy subjects (19–24). Until today, only our group have explored relevant molecular biomarkers potentially responding to a classic psychedelic, specifically to ayahuasca, in MDD patients (25–27).

Ayahuasca is an Amazonian brew made with *Psychotria viridis*, a plant with N,N-Dimethyltryptamine (N,N-DMT) with agonist interaction with 5HT-2A receptors and σ1 receptors, and *Banisteriopsis caapi* a vine that contains β-carbolines (tetrahydroharmine, harmaline, and harmine) which are reversible monoamine oxidase inhibitors (MAOi) (28–31). Ayahuasca’s multi-target activity on pathways related to MDD is associated with no observations of dose-resistance, physiological or psychological dependence, cognitive impairment, physiological toxicity, or long-lasting adverse effects (32, 33). This has encouraged experimental studies on cognition and mood in healthy ayahuasca users (15, 20, 34–38), as well as some clinical trials testing ayahuasca for treatment-resistant depression, which to date have shown positive antidepressant results (8, 39–41).

In our previous randomized placebo-controlled trial (RCT), patients with treatment-resistant depression were dosed once with ayahuasca or placebo, with a significant and increasing antidepressant effect induced by ayahuasca being observed one, two and seven days after intervention (8). Furthermore, suicidal ideation and attempts were also reduced along a week following ayahuasca intake (41).

In that same RCT, key molecular biomarkers of major depression (MDD) were analyzed at baseline and two days after the intervention to aim to better understand the biological antidepressant action of ayahuasca (42, 43). In sum, the results revealed that at baseline patients presented hypocortisolemia, blunted salivary cortisol awakening response (CAR) and a low-grade pro-inflammatory profile [shown by increased C-Reactive Protein (CRP) levels] (25–27). However, after ayahuasca intake, but not for placebo, those patients had their CAR and CRP levels improved (26, 27). An increase in plasma Brain-derived Neurotrophic Factor (BDNF) levels was also observed. It is interesting to highlight that both BDNF and inflammation improvements were correlated with reductions in depressive symptoms assessed 2 days after dosing (25, 27).

Brain-derived Neurotrophic Factor is a neurotrophin that has been related to antidepressant response. It is believed that part of the antidepressant action is related to a recovery in neurogenesis and neuroplasticity processes, as well through an anti-inflammatory response (44). Usually, the studies regarding the alacrity of effect from psychedelics agents in neurogenesis and neuroplasticity and their anti-inflammatory response derives from preclinical research with *in vitro* and rodents’ models (45–47).

In addition to the investigation regarding efficacy and effectiveness of pharmacological agents in RCTs, conditional process analyses could also offer a new perspective to such studies, refining the causal relationship between a treatment and its outcomes, establishing variables that can potentiate treatment or better understanding the mechanisms of how efficacy is achieved (48, 49). This statistical approach includes mediation and moderation analyses. The mediator is a variable indirectly involved in the link between the treatment and the effect. In turn, a variable is called a moderator when an effect is observed only in certain levels of this variable. Moderators interact with the independent variable to modify its link with the effect, that is, they determine in what circumstances the effect is observed (48, 49). Investigating moderators of treatment may improve the statistical power of RCTs by helping better define the inclusion/exclusion criteria (48).

Therefore, herein we report in what circumstances acute emotional (depressive symptoms), cognitive (psychedelic experience), and physiological parameters (salivary cortisol) collected during ayahuasca and placebo dosing session in that RCT (8) moderated certain key MDD molecular biomarkers (serum BDNF, serum cortisol, serum interleukin 6, plasma CRP, and CAR) two days after intervention in patients with treatment-resistant depression versus a group of healthy volunteers. We hypothesized that acute parameters assessed during the dosing session (cortisol, clinical depressive response, and psychedelic experience) would moderate the improvement in the post-treatment MDD biomarkers only for patients of the ayahuasca group. Specifically, we expected to find biomarker changes only for patients who showed an acute raised cortisol level, a stronger antidepressant response, and a greater psychedelic experience resulting from ayahuasca intake.

## Materials and methods

This study is part of a RCT that investigated the antidepressant effects of ayahuasca for treatment-resistant depression (8). This study was conducted at the Onofre Lopes University Hospital (HUOL) of the Federal University of Rio Grande do Norte (UFRN), Brazil. It was registered at http://clinicaltrials.gov (NCT02914769, registered 23/09/2016) and approved by the Onofre Lopes University Hospital Ethics Committee for Medical Research (CEP/HUOL) (#579.479). Moreover, this study fulfills the ethical standards of the relevant national and institutional committees for human experimentation and with the Declaration of Helsinki of 1975, revised in 2008. All patients provided a written consent prior to their participation.

This study is registered at http://clinicaltrials.gov under the name “Antidepressant Effects of Ayahuasca: A Randomized Placebo Controlled Trial in Treatment Resistant Depression” (NCT02914769).^1^

### Volunteers

The 72 volunteers, patients (P; *n* = 28) and healthy controls (C; *n* = 44), adults (18–60 years old) and naïve to any classic serotonergic psychedelic (ayahuasca, LSD, psilocybin, and mescaline), participated in the trial. The control group comprised healthy adult volunteers, who met the listed inclusion criteria: no present diagnosis or history of neuropsychiatric diseases, no current inflammatory or metabolic diseases or pregnancy, and not receiving current medications with effects on cognitive, mood, neurovegetative, immune, or endocrine function. Patients selected were in a current moderate-to-severe depressive episode at screening (Hamilton Depression Scale HAM-D 17) and had diagnosed treatment-resistant depression. Treatment-resistance was defined as having an inadequate response, that is, not achieving remission, after at least two treatments with antidepressant medications from different classes (50). For patients, the exclusion criteria were: pregnancy, current or previous history of neurological disorders, personal or family history of schizophrenia, bipolar affective disorder, mania or hypomania, substance use-related disorder, and suicidal risk.

All volunteers (patients and healthy controls) had a full clinical mental health evaluation and anamneses by a trained psychiatrist to assure the inclusion and exclusion criteria was abided by. The Structured Clinical Interview for Axis I (SCID; ESM-IV) was administered for all volunteers (healthy control and patients) to assess the inclusion/exclusion criteria. For patients, the SCID was also used to confirm the unipolar major depressive disorder diagnosis, while the severity of symptoms were assessed by the Montgomery–Åsberg Depression Rating Scale (MADRS) (51). During this study all patients underwent a 2-week washout period for changing of antidepressant, so they were antidepressant free (since it is important to avoid a potential serotonergic interaction with ayahuasca). This washout period could be slightly adjusted with respect to half-life of patient previous medications.

### Study design

**FIGURE 1:**
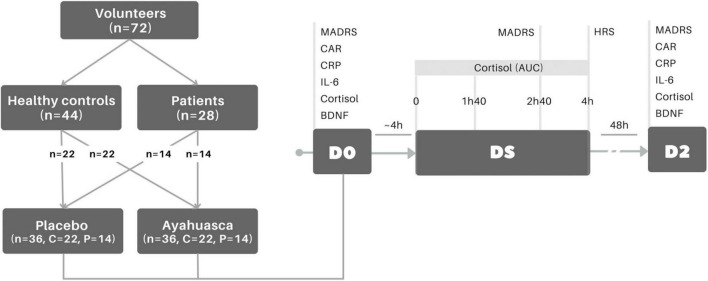
Experimental design. Groups comprised treatment-resistant patients (*n* = 28) and healthy controls (*n* = 44). Each group had half of their participants allocated to a treatment: placebo (*n* = 36, P: *n* = 14, C: *n* = 22) or ayahuasca (*n* = 36, P: *n* = 14, C: *n* = 22). At baseline (D0), MADRS was assessed to quantify depressive symptoms, as well as blood and saliva samples were collected to measure biochemical markers. At the dosing session (DS), when the volunteer (patients or healthy control) takes ayahuasca or placebo, saliva samples were collected at times 0, 1 h40, 2 h40, and 4 h to determine AUC salivary cortisol and MADRS was assessed at 2 h40. After the dosing session (4 h), participants filled HRS to evaluate hallucinogenic experience. Two days after dosing session (D2 ∼48 h), MADRS, blood and saliva were collected again. MADRS, Montgomery–Åsberg Depression Rating Scale; CAR, salivary cortisol awakening response; CRP, plasma C-reactive protein; IL-6, serum interleukin 6; BDNF, serum brain-derived neurotrophic factor; HRS, hallucinogenic rating scale; AUC, area under the curve.

Patients and controls were treated with a single oral liquid dose of ayahuasca or placebo, with a randomization ratio 1:1, in a parallel two arms design (8). From those 28 patients and 44 healthy controls, half of them received ayahuasca (P = 14, C = 22) and the other half placebo in the dosing session (P = 14, C = 22) (Figure 1). Volunteers were individually taken for medical appointments, blood collection and dosing sessions, to avoid social interaction between them, reducing social learning and the substance-related expectation. The setting was uniform for all participants since the medical appointments and dosing sessions were always performed in the same rooms of the psychiatry unit of the University Hospital (HUOL). During dosing session, a psychiatrist and three researchers were available to provide support if needed in a side room separated by a glass wall. All members of the study did not interact with patients during the dosing session unless they needed help or during data assessment. The experimental session lasted about 8 h, starting always at 10 am, independent of whether the substance (ayahuasca or placebo), although ayahuasca effects usually lasted 4 h. The same instructions regarding study methods, and possible substance effects were given to all participants. It was informed that they could experience something or nothing about feelings or sensations either intaking ayahuasca or placebo. The participants spent the session sitting in a comfortable reclining armchair and could use a blanket to cover up. While under the influence of ayahuasca or placebo, the volunteers were oriented to stay with their eyes closed (no blindfold was provided) and focus on their body, mind and in the music. The tracks, played to participants via headphones, comprised of instrumental music and traditional songs in Brazilian Portuguese (Ayahuasca Therapeutic Playlist, by LF Tofoli, at Spotify). During the dosing session, salivary cortisol and some psychometric instruments (described below) were collected. Blood and saliva samples were collected at baseline (D0), approximately 4 h before the dosing session, and 2 days after the dosing session (D2). Different blinded psychiatrists were present for screening, dosing session and follow-up (48 h) aiming to reduce expectations related to treatment response. For more detail cf. (8) (Figure 1).

### Ayahuasca

A single oral liquid dose of 1 ml/kg of ayahuasca was administrated per volunteer. A single batch was used in the entire RCT and its component’s concentrations were dosed at two different time points by mass spectroscopy analysis to assure their stability, resulting in the following values: 0.36 ± 0.01 mg/ml DMT, 1.86 ± 0.11 mg/ml harmine, 0.24 ± 0.03 mg/ml harmaline, and 0.20 ± 0.05 mg/ml THH. This batch was prepared by a Barquinha church, at Ji-Paraná, Rondônia-Brazil. For more detail of its preparation please see Galvão-Coelho et al. (27).

### Placebo

The placebo was made of water, yeast, citric acid, caramel colorant, and zinc sulphatean, resulting in a similar aspect to the taste and color of the ayahuasca brew. Although no psychoactive effect was induced by the placebo, it could simulate some of the physical sensations experienced during ayahuasca intake, such as nausea, vomiting, and diarrhea (8). Since all participants were naïve for any psychedelic experience, we expected that such physical effects could assist in maintaining participant blindness with respect to the substance intake.

### Molecular biomarkers

Saliva samples were collected thrice during the dosing session: 1 h40, 2 h40, and 4 h after the beginning of ayahuasca intake. Moreover, at D0 and D2 three saliva samples were obtained at 0, 30 and 45 min after awakening, usually about 6 am, in order to measure the CAR. During these 45 min, the volunteer stayed at rest and in 8 h of fasting. Saliva samples were collected using a specific cotton stick, Salivette (Sarstedt, Germany).

At D0 and D2, after saliva collection, blood samples were collected at rest and after an 8 h-fasting, to assess plasma CRP and serum interleukin 6 (IL-6), cortisol, and BDNF. The IL-6, BDNF and cortisol were measured by ELISA (IL-6: ELISA BD 20 IL-6 HU (PMG), BDNF: Merck Millipore, serum cortisol: DGR-SLV 1887, and salivary cortisol: DGR-SLV 4635), and CRP by immunoturbidimetry. All biomarkers were dosed in duplicate and blinded for each volunteer.

### Psychometric instruments

Assessment of MADRS was conducted at D0 and D2. Patients that presented total MADRS score in D2 ≤ 10 were in remission (52), while a reduction of 50% in depressive symptoms assessed by MADRS between D2 and D0 was considered as treatment response. Given that MADRS is one of the most used tools to assess depressive symptoms changes in short intervals, it was selected to assess symptoms changes during the dosing session (53, 54). Therefore, during the dosing session MADRS was evaluated before and at 1 h40, 2 h40, and 4 h after ayahuasca/placebo intake. We chose to use the assessment at 2 h40 as the acute outcome in the present study, since it is when the peak of ayahuasca effects typically occurs (36).

The Hallucinogenic Rating Scale (HRS) (55) was assessed after dosing when the acute psychedelic effects of ayahuasca was estimated to ceased. It is a sensitive tool for measuring the effects of classic psychedelics, and is usually assessed in clinical trials with psychedelics (56–58). The HRS is composed by 6 factors: intensity, somaesthesia, affect, perception, cognition, and volition.

### Statistical analysis

For CAR and the four samples of salivary cortisol collected during the dosing session, the area under the curve (AUC) was calculated separately and used in statistical analyses.

Simple moderation analysis was applied to investigate potential moderators (*W*) of the relationship between the dependent (*Y*) and independent (*X*) variables for ayahuasca and placebo ingestion (59). A significant interaction means that the relationship between the dependent and independent variables (X*Y) is somehow changed in the presence of the moderator. A follow-up analysis was then performed to assess the slope of this relationship at different levels of the moderator: low (1 standard deviation below the mean) and high (1 standard deviation above the mean).

Herein, the moderators (*W*) investigated were: the AUC of salivary cortisol, changes in MADRS from D0 to 2h40 of the dosing session (ΔMADRS_D0–2h40_) and the HRS total score, all of them collected during the experimental session with ayahuasca (*n* = 36) or placebo (*n* = 36). The dependent variables (*Y*) were the following MDD molecular biomarkers at D2: CAR, serum BDNF, serum cortisol, serum IL-6 and plasma CRP. The independent variables (*X*) used were group (Control and Patient; treated as dummy variable and set to 0 and 1, respectively). Then, the patients’ clinical response (MADRS_D0_ − MADRS_D2_) was used as independent variables (*X*) for those groups (P/C) and substances (ayahuasca/placebo) that have showed significant results at the first step analysis. The standardized residuals for each main model (X*Y) were evaluated for normality (Shapiro–Wilk test), and only the models with normally distributed residuals had their follow-up analysis considered.

For all tests, the molecular biomarkers were log-transformed. The analyses were not controlled for age and sex as a limitation of the package used for the analysis. The statistical significance was set to *p* ≤ 0.05 and confidence intervals (95%) are shown for estimates on Supplementary Tables. The analyses were performed in R (version 4.0.2), using the package *processr* (version 0.0.0.9000).

## Results

### Sample characteristics and main ayahuasca/placebo effects

The consolidated standards for clinical trial reports (CONSORT) are shown in Supplementary Figure 1. From a total of 328 pre-screened volunteers, 28 patients with treatment-resistant depression (P) and 44 healthy controls volunteers (C) were analyzed. A control volunteer allocated to placebo group was removed from the analyses because of lacking some biomarker measures data. All volunteers were adult Brazilians. Both groups had the same proportion of sex [Chi-square test: Ayahuasca: P = 78.6%, C = 54.5%, χ^2^(1) = 1.26, *p* = 0.27; Placebo: P = 71.4%, C = 59.1%, χ^2^(1) = 0.16, *p* = 0.69], and homogeneous ages [ANOVA: group × treatment: *F*_(1,68)_ = 0.56, *p* = 0.457]. Furthermore, patients had lower income and education than controls. Patients were in a current episode of severe major depression (HAM-D = 21.57 ± 5.27, MADRS = 32.68 ± 6.32), with about 10.71 ± 9.72 years of disease and 3.07 ± 1.59 depressive episodes (Supplementary Table 1).

The main RCT result where ayahuasca outperformed placebo in its antidepressant action was previously published by our group [Between-groups effect size was large at D1 (24 h after dosing; Cohen’s *d* = 0.84; 95% CI 0.05–1.62) and D2 (48 h after dosing; Cohen’s *d* = 0.84; 95% CI 0.05–1.63] (8). The SC, CAR, BDNF, IL-6, and CRP responses 48 h (D2) after ayahuasca dosing, comparing to placebo, were also previously published by our group, as well as salivary AUC cortisol changes along dosing session (25–27).

### Moderation analyses of acute parameters on MDD biomarkers

**FIGURE 2:**
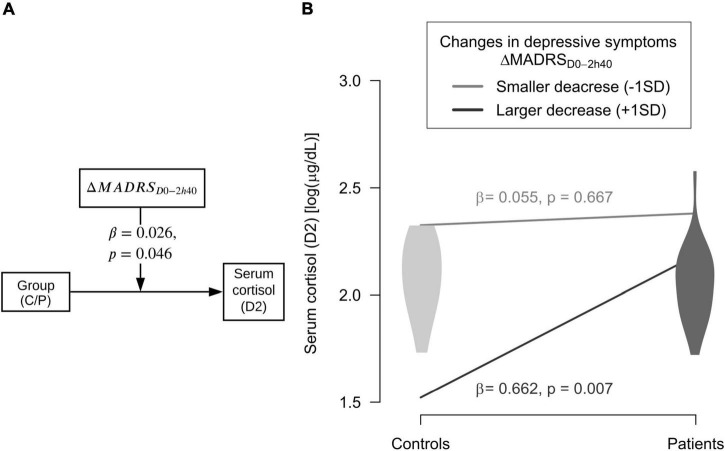
Moderator of ayahuasca antidepressant action. **(A)** Changes in depressive symptoms between baseline and 2 h40 after ayahuasca intake (ΔMADRS_D0–2h40_) moderated the significant relation between groups (C = controls; P = patients) and serum cortisol levels at D2 (2 days after dosing). **(B)** Larger decreases in depressive symptoms moderated higher serum cortisol levels of patients at D2, but not for controls. Dark gray line and violin plot mean statistically significant interaction (*p* ≤ 0.05); light gray line and violin plot mean not statistically significant interaction.

Changes in depressive symptoms between D0 and 2h40m during ayahuasca dosing session (ΔMADRS_D0–2h40_) moderated the relationship between group (P/C) and serum cortisol at D2 [*F*_(3,32)_ = 4.145, *R*^2^ = 0.280, β = 0.026, *p* = 0.046]. Specifically, larger acute decreases in depressive symptoms scores were related to higher levels of serum cortisol only for patients at D2, and not for controls (β = 0.662, SE = 0.230, *t* = 2.881, *p* = 0.007) (Figure 2 and Supplementary Table 2).

No significant moderator of ayahuasca effects were found for serum BDNF, CAR, or plasma CRP levels at D2, both for patients and healthy controls (Supplementary Table 2). Moreover, no moderator of placebo effects on biomarkers (D2) was found for the patients vs healthy controls analyses (Supplementary Table 3).

**FIGURE 3:**
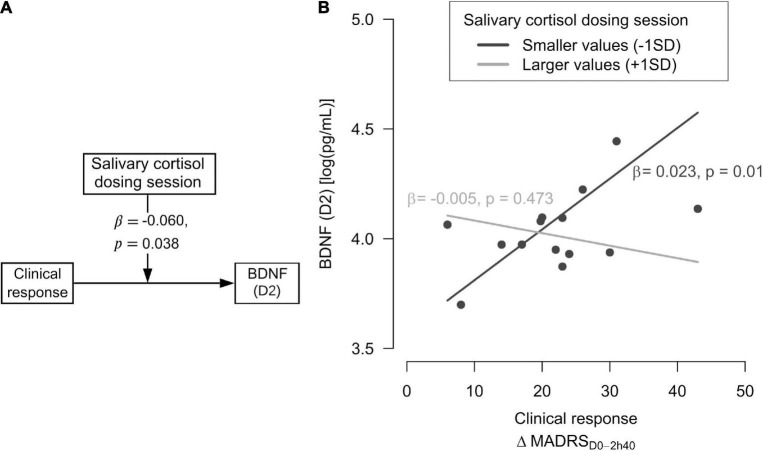
Moderator of ayahuasca antidepressant action according to patients’ clinical response. **(A)** The salivary cortisol measured at the dosing session moderated the relationship between clinical response (MADRS_D0_-MADRS_D2_) and BDNF levels at D2 (2 days after dosing). **(B)** Smaller salivary cortisol changes were associated with higher serum Brain-derived Neurotrophic Factor (BDNF) levels in patients with stronger clinical response (reductions in depressive symptoms). Dark gray line means statistically significant interaction (*p* ≤ 0.05); light gray line means not statistically significant interaction.

Since only patients who ingested ayahuasca, and not placebo or healthy control group, showed a significant moderator of its biological response, a posterior analysis was made only for patients that were randomized to ayahuasca (aiming to better understanding these biological reactions regarding the patient’s clinical response). Results revealed that acute changes in salivary cortisol levels during ayahuasca dosing session significantly moderated the relationship between the patients’ clinical response (MADRS_D0_ − MADRS_D2_) and BDNF levels at D2 [*F*_(3,10)_ = 3.48, β = −0.060, *R*^2^ = 0.511, *p* = 0.038]. More precisely, the positive correlation between clinical response and D2 BDNF levels only happened for those patients who showed small increases of cortisol during the experimental session. (β = 0.023, SE = 0.007, *t* = 3.16, *p* = 0.01) (Figure 3).

No significant moderators of ayahuasca biological antidepressant action were found for interactions between patients’ clinical response (MADRS_D0_ − MADRS_D2_) and CAR, serum cortisol, plasma CRP, or IL-6 (Supplementary Table 4).

Total HRS had no moderating role for biomarkers both for group (Patients/Control) and patient’s clinical response analyses (Supplementary Tables 3, 4).

## Discussion

As we expected, some acute measures assessed during ayahuasca dosing, and not placebo, moderated the improvements of MDD biomarkers two days after the session in patients with treatment-resistant depression. This also did not occur for the control group. More specifically, larger acute decreases of depressive symptoms moderated higher levels of serum cortisol in those patients. While lower acute changes in salivary cortisol levels were related to higher BDNF levels in patients with a larger clinical response.

The investigations made here comparing ayahuasca and placebo response in patients and healthy volunteers are important because as these groups of subjects have distinctly mental and physical functioning, different patterns of neurobiological responses to these substances might be observed, as actually were seen. The same importance is regarded for the placebo control group used in this study, despite the accepted challenges with blinding in psychedelic field research (60).

Changes in perception, cognition and emotion usually start around 40 min after ayahuasca intake, when an increased introspection allows the awareness of a flow of visual effects, autobiographical and emotional memories (15, 34, 61–64). It is hypothesized that during this mental flow, knowledge is gained by insights and the enhanced internal morals contribute to later behavioral change (65). Some studies have identified a positive relationship between the cognitive psychedelic experience and clinical response in mood and anxiety outcomes, as well as for substance abuse. For our group of patients with treatment-resistant depression, larger hallucinogenic perceptions during the ayahuasca experience were related to a greater reduction in depressive symptoms seven days after dosing, as was showed in our previous study (8). The occurrence of mystical experiences was also described as a possible mediator of psilocybin antidepressant response (56, 57). Furthermore, recent online surveys reported that perceived mystical experience and number of “insights” during an ayahuasca ceremony were beneficial in the improvement of depressive symptoms (6). Surprisingly, HRS did not moderate any biomarkers either in the group or in the clinical response analysis.

Despite some studies having explored the relationship between mystical and hallucinogenic experience during the psychedelic state and its relationship with antidepressant response, until now no study has explored what factors that occur during psychedelic state modulate biological response associated with the antidepressant action of these substances in patients with major depression. The results that we have shown here address this prior shortfall.

Larger acute reductions in depressive symptoms (between initial state and 2 h40 m of the experimental session) were statistically significant for improvement of D2 cortisol only for MDD patients that ingested ayahuasca, and not for controls. It is important to highlight that at baseline our patients displayed a markedly hypocortisolemic profile (26), hence this increase in cortisol levels moderated by acute reductions in depressive symptoms has a potential therapeutic value and may assist this particular phenotype of patient. This may allow for the provision of modulating homeostatic cortisol levels, thereby assisting in adjusting one of the main biomarkers associated with MDD (42, 43).

Moreover, in this study, smaller changes in salivary cortisol levels during the dosing session moderated higher BDNF levels at D2 in patients with a larger clinical response (indicating a larger reduction in depressive symptoms between D0 and D2). On the other hand, this relationship was not observed for patients with weak clinical response. Both BDNF expression and its physiological role are modulated by cortisol through the promoter region of the BDNF gene and its TrkB receptor, respectively. Interestingly, an inverted U-curve relationship has been observed between cortisol and BDNF levels, where high and low cortisol levels are implicated in reduced gene expression and high cortisol levels also reduce the potential response of BDNF receptor (66, 67). We emphasize that the rise in BDNF levels induced by ayahuasca two days after dosing, which was showed in our previous study (25), was associated with a clinical antidepressant response. We can now further contend that this antidepressant BDNF response was mainly modulated by acute cortisol changes during ayahuasca intake. Few studies have investigated BDNF response to classic psychedelics in humans, with some revealing that LSD seems to have a biphasic action on BDNF plasma levels, the low (5 and 20 μg) and high doses (200 μg) (21) induce acute increases in BDNF levels of healthy subjects compared to that for the placebo (23, 68), and no changes were observed after a medium LSD dose intake (100 μg) (69).

We did not find significant modulatory effects of ayahuasca on CRP and IL-6 levels. The previous study that analyzed this cytokine in our ayahuasca RCT did not find significant changes for blood IL-6 as response to this psychedelic, thus no significant moderator was expected for this biomarker (27). However, a study that has investigated inflammation biomarkers response to a classic psychedelic observed a decrease of salivary IL-6 levels in healthy subjects after a single inhalation of 5-MeO-DMT (24). On the other hand, our patients dosed with ayahuasca, but not placebo, decreased their CRP levels, which was correlated with a decreased depressive symptoms (27). Surprisingly none of these acute parameters assessed during ayahuasca dosing session seems to be important to this anti-inflammatory response.

It is important to point out some limitations of the present study, such as a single blood and saliva sample per volunteer before and after intervention, which is recognized as not designed to detect the multiple molecular biomarkers of response. In fact, such single sampling may have limited the find of moderators for ayahuasca anti-inflammatory response. We also analyzed the total BDNF levels instead of mature-BDNF and pro-BDNF, which have a more specific action than the BDNF. Therefore, future studies should assess longer-term response of biomarkers to certify if they have the same long-lasting effect of antidepressant response (4), as well as considering specific BDNF isoform analysis, for a better understanding of MDD neurobiology and therapeutics. We also encourage the investigation of the biomarkers’ mediator effect, to shed light on the biochemical mechanisms of ayahuasca antidepressant actions.

One of the greatest challenges in psychedelic trials is the adequate blinding of participants in placebo-controlled studies. To address this, some important actions have been implemented in the studies from our group. Regarding our choice of placebo, although it still not been the ideal (due to lacking a psychoactive effect), studies have used some active substances that simulate some psychological effects, such as a minor sedation induced by an antihistamine drug, and/or physical effects of the psychedelic (4, 60). Aiming to reduce the participant’s expectation about treatment, and placebo response, some cautions must be attended to about personal experiences, observational learning, instructions from staff, and clinical setting. The placebo used in our study was physically active and it could simulate nausea, vomiting, and diarrhea like ayahuasca does. Moreover, all participants had no previous experience with any psychedelics, which was a factor contributing to the successful blinding, and additionally we reduced volunteer’s treatment expectation (with the same instructions regarding substance effects given to all volunteers). In addition, all participants individually underwent the same procedures thereby avoiding social learning. The settings were uniform and different blinded psychiatrists were along the study.

Advancing previous reports that showed the importance of subjective psychedelic experience to clinical response (6, 8, 70), this work corroborates this data and provides a step-forward as a pioneering psychedelic field study assessing the biological changes of MDD molecular biomarkers. While we found a relationship between, first: acute mood changes and serum cortisol, and second: acute changes in depressive symptoms and BDNF of antidepressant biological ayahuasca response, we must consider that in this context all processes and relations were induced by both a complex interaction between pharmacological action and alteration in consciousness.

In sum, acute emotional (depressive symptoms reduction) and physiological (lower salivary cortisol) effects of ayahuasca intake seem to be relevant to an improvement of key MDD molecular biomarker (namely serum cortisol and BDNF). This effect is observed in patients with depression rather than in healthy controls and in those patients with higher clinical response. These results pave the way for future studies focusing on the biological and psychological changes as a result of psychedelic therapies.

## Data availability statement

The original contributions presented in this study are included in the article/Supplementary material, further inquiries can be directed to the corresponding author.

## Ethics statement

The studies involving human participants were reviewed and approved by the Onofre Lopes University Hospital Ethics Committee for Medical Research. The patients/participants provided their written informed consent to participate in this study.

## Author contributions

NG-C, EN, JM-d-O, FP-F, BL-S, and DA conducted the clinical trial. AM and RA performed the dosing of biomarkers. GS and VO carried out the statistical analyses. All authors drafted and edited the manuscript.

## Footnotes

1
https://clinicaltrials.gov/ct2/show/NCT02914769?term=NCT02914769&draw=2&rank=1
