# Supplementary material for: Moderators of ayahuasca’s biological antidepressant action
Source: Front Psychiatry. 2022 Dec 5;13:1033816. doi: 10.3389/fpsyt.2022.1033816 (PMC9760741; doi:10.3389/fpsyt.2022.1033816)
Supplement: Supplementary file 1 [file Image_1.pdf]

Biological Moderators of Ayahuasca's Antidepressant Action

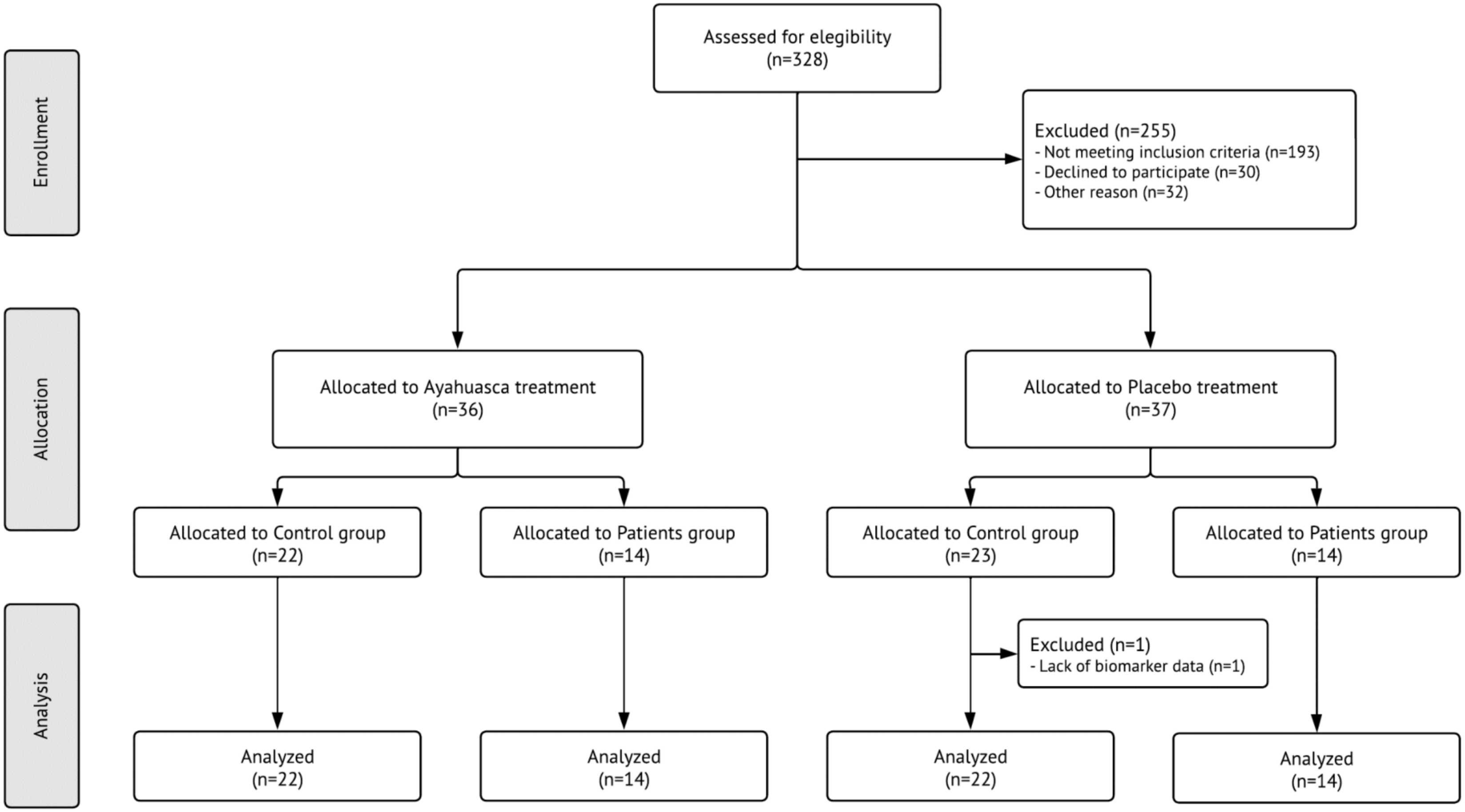

Figure S1. The consolidated standards of clinical trial reports (CONSORT) for control group (C) and treatment-resistant depressive patients (P) allocated to either an ayahuasca or a placebo treatment in a double-blind placebo control clinical trial.
