# Supplementary material for: Moderators of ayahuasca’s biological antidepressant action
Source: Front Psychiatry. 2022 Dec 5;13:1033816. doi: 10.3389/fpsyt.2022.1033816 (PMC9760741; doi:10.3389/fpsyt.2022.1033816)
Supplement: Supplementary file 2 [file Table_1.pdf]

Table S1. Sociodemographic and clinical characteristics of the sample.

		Ayahuasca group		Placebo group	
		**Control**	**Patients**	**Control**	**Patients**
**Participants**	*n*	22	14	22	14
**Sex**	*Women*	12 (54.5%)	11 (78.6%)	13 (59.1%)	10 (71.4%)
	Men	10 (45.5%)	3 (21.4%)	9 (40.9%)	4 (28.6%)
**Age (years)**	*Mean (SD)*	31.0 (8.8)	39.9 (11.4)	30.6 (10.4)	43.3 (12.0)
**Education**	*Undergraduate*	9 (40.9%)	11 (78.6%)	17 (77.3%)	12 (85.7%)
	Graduated	13 (59.1%)	3 (21.4%)	5 (22.7%)	2 (14.3%)
**Income**	*Low*	8 (36.4%)	10 (71.4%)	13 (59.1%)	12 (85.7%)
	High	14 (63.6%)	4 (28.6%)	9 (40.9%)	2 (14.3%)
**HAM-D**	*Mean (SD)*	0.29 (0.63)	24.1 (5.34)	0.77 (1.15)	19.1 (3.95)
**MADRS**	*Mean (SD)*	0.19 (0.50)	36.1 (6.13)	0.68 (1.29)	29.2 (4.42)
**Years of disease**	*Average*	-	8.79 (6.25)	-	12.6 (12.2)
**Number of episodes**	*Average*	-	2.71 (1.33)	-	3.43 (1.79)

HAM-D= Hamilton Depression Rating Scale for Depression; MADRS= Montgomery-Asberg Depression Rating Scale.
