# Supplementary material for: Moderators of ayahuasca’s biological antidepressant action
Source: Front Psychiatry. 2022 Dec 5;13:1033816. doi: 10.3389/fpsyt.2022.1033816 (PMC9760741; doi:10.3389/fpsyt.2022.1033816)
Supplement: Supplementary file 3 [file Table_2.pdf]

**Table S2**. Statistical values of interaction (β) and $R^2$ of moderation analyses of acute physiological (AUC= Area Under the Curve of salivary cortisol), cognitive (HRS= Hallucinogenic Rating Scale) and emotional outcomes ($\Delta MADRS_{D0\text{-}2h40}$ = changes in MADRS from D0 to 2h40 of the dosing session) during ayahuasca experimental session on serum Brain-derived Neurotrophic Factor (BDNF), serum cortisol (SC), salivary cortisol awakening response (CAR), plasma C-Reactive protein (CRP) and serum interleukin 6 (IL-6), two days after treatment (D2) on treatment-resistant depressive patients and healthy control groups.

| | **BDNF** | | **SC** | | **CAR** | | **CRP** | | **IL-6** | |
|---|---|---|---|---|---|---|---|---|---|---|
| | β | $R^2$ | β | $R^2$ | β | $R^2$ | β | $R^2$ | β | $R^2$ |
| Group*AUC | -.099 | .054 | -.115 | .007 | .734 | .245 | 1.115 | .168 | 1.395 | .707 |
| | (-.558, .360) | | (-.818, .589) | | (-.232, 1.700) | | (-7.390, 5.161) | | (-2.095, 4.885) | |
| Group*$\Delta MADRS_{D0\text{-}2h40}$ | -.004 | .145 | **.026** | **.280** | -.006 | .013 | .067 | .235 | -.041 | .727 |
| | (-.023, .014) | | **(.000, .052)** | | (-.054, .041) | | (-.192, .325) | | (-.186, .103) | |
| Group*HRS | -.012 | .031 | -.021 | .036 | .017 | .013 | .173 | .176 | .233 | .726 |
| | (-.051, .026) | | (-.079, .037) | | (-.075, .109) | | (-1.329, 2.01) | | (-.048, .514) | |

Bold values stand for significant interactions. Values inside brackets represents the 95% confidence interval for the estimate (β). $SC_{ES}$= salivary cortisol collected during the experimental session, $\Delta \mathit{MADRS}_{D0\text{-}2h40}$= changes in Montgomery–Åsberg Depression Rating Scale (MADRS) from 4h before the experimental session (D0) until 2h40 of experimental session; $\Delta MADRS_{ES\text{-}2h40=}$ changes in MADRS from baseline until the 2h40 of experimental session; HRS= Hallucinogenic Rating Scale.
