# Supplementary material for: Moderators of ayahuasca’s biological antidepressant action
Source: Front Psychiatry. 2022 Dec 5;13:1033816. doi: 10.3389/fpsyt.2022.1033816 (PMC9760741; doi:10.3389/fpsyt.2022.1033816)
Supplement: Supplementary file 4 [file Table_3.pdf]

Table S3. Placebo experimental session: Statistical values of interaction (β) and R^2 of moderation analyses of acute physiological (AUC= Area Under the Curve of salivary cortisol), cognitive (HRS= Hallucinogenic Rating Scale) and emotional outcomes ($\Delta MADRS_{D0-2h40}$ = changes in MADRS from D0 to 2h40 of the dosing session) during placebo experimental session on serum Brain-derived Neurotrophic Factor (BDNF), serum cortisol (SC), salivary cortisol awakening response (CAR), plasma C-Reactive protein (CRP) and serum interleukin 6 (IL-6), two days after placebo intake (D2) on treatment-resistant depressive patients and healthy control groups.

	**BDNF**		**SC**		**CAR**		**CRP**		**IL-6**	
	β	R^2	β	R^2	β	R^2	β	R^2	β	R^2
Group*AUC	.000	.059	.000	.042	.001	.313	-.008	.240	.009	.123
	(-.002, .001)		(-.003, .002)		(-.001, .004)		(-.031, .016)		(-.011, .029)	
Group*$\Delta MADRS_{D0-2h40}$	-.035	.133	.006	.070	.009	.225	-.051	.202	.390	.091
	(-.077, .007)		(-.058, .070)		(-.078, .096)		(-.752, .651)		(-.204, .983)	
Group*HRS	.051	.253	-.116	.183	-.176	.285	-.051	.489	.455	.019
	(-.036, .139)		(-.248, .017)		(-.363, .010)		(-2.283, .220)		(-.920, 1.829)	

Bold values stand for significant interactions. Values inside brackets represents the 95% confidence interval for the estimate (β). SC_{ES}= salivary cortisol collected during the experimental session, $\Delta MADRS_{D0-2h40}$= changes in Montgomery–Åsberg Depression Rating Scale (MADRS) from 4h before the experimental session (D0) until 2h40 of experimental session; $\Delta MADRS_{ES-2h40}$= changes in MADRS from baseline until the 2h40 of experimental session; HRS= Hallucinogenic Rating Scale.
