# Supplementary material for: Moderators of ayahuasca’s biological antidepressant action
Source: Front Psychiatry. 2022 Dec 5;13:1033816. doi: 10.3389/fpsyt.2022.1033816 (PMC9760741; doi:10.3389/fpsyt.2022.1033816)
Supplement: Supplementary file 5 [file Table_4.pdf]

Table S4. Statistical values of interaction (β) and R^2 of moderation analyses of acute physiological, (AUC= Area Under the Curve of salivary cortisol), cognitive (HRS= Hallucinogenic Rating Scale) and emotional outcomes ($\Delta MADRS_{D0\text{-}2h40}$ = changes in MADRS from D0 to 2h40 of the dosing session) during ayahuasca experimental session on serum Brain-derived Neurotrophic Factor (BDNF), serum cortisol (SC), salivary cortisol awakening response (CAR), plasma C-Reactive protein (CRP) and serum interleukin 6 (IL-6), two days after treatment (D2) on clinical response ($MADRS_{D0}$ – $MADRS_{D2}$) of patients with treatment-resistant depression.

	**BDNF**		**SC**		**CAR**		**CRP**		**IL-6**	
	β	R^2	β	R^2	β	R^2	β	R^2	β	R^2
Response*AUC	**-.060**	**.511**	-.022	.272	.082	.333	-.429	.201	-.153	.032
	**(-.116, -.004)**		(-.105, .061)		(-.096, .259)		(-1.398, .539)		(-.854, .547)	
Response*$\Delta MADRS_{D0\text{-}2h40}$	.000	.341	.000	.272	.001	.105	-.005	.174	-.002	.215
	(-.001, .000)		(-.001, .001)		(-.001, .004)		(-.018, .009)		(-.010, .007)	
Response*HRS	.007	.38	.005	.163	.018	.139	-.048	.164	.027	.102
	(-.004, .018)		(-.009, .020)		(-.017, .054)		(-.222, .126)		(-.091, .146)	

Bold values stand for significant interactions. Values inside brackets represents the 95% confidence interval for the estimate (β). SC dosing session= salivary cortisol collected during the experimental session, $\Delta MADRS_{D0\text{-}2h40}$= changes in Montgomery–Åsberg Depression Rating Scale (MADRS) from baseline (D0) until 2h40 of experimental session; HRS= Hallucinogenic Rating Scale.
